# Serum IgG anti-SARS-CoV-2 Binding Antibody Level Is Strongly Associated With IgA and Functional Antibody Levels in Adults Infected With SARS-CoV-2

**DOI:** 10.3389/fimmu.2021.693462

**Published:** 2021-10-08

**Authors:** Xunyan Ye, Laura S. Angelo, Erin G. Nicholson, Obinna P. Iwuchukwu, Wanderson Cabral de Rezende, Anubama Rajan, Letisha O. Aideyan, Trevor J. McBride, Nanette Bond, Patricia Santarcangelo, Yolanda J. Rayford, Laura Ferlic-Stark, Sonia Fragoso, Zoha Momin, Hongbing Liu, Khanghy Truong, Brianna Lopez, Margaret E. Conner, Andrew P. Rice, Jason T. Kimata, Vasanthi Avadhanula, Pedro A. Piedra

**Affiliations:** ^1^ Department of Molecular Virology & Microbiology, Baylor College of Medicine, Houston, TX, United States; ^2^ Department of Pharmacology, Baylor College of Medicine, Houston, TX, United States; ^3^ Department of Pediatrics, Baylor College of Medicine, Houston, TX, United States

**Keywords:** SARS-CoV-2, COVID-19, serology, human serum, binding antibody, functional antibody

## Abstract

**Background:**

Severe acute respiratory syndrome coronavirus 2 (SARS-CoV-2) was first reported in December 2019 in Wuhan, China, and then rapidly spread causing an unprecedented pandemic. A robust serological assay is needed to evaluate vaccine candidates and better understand the epidemiology of coronavirus disease (COVID-19).

**Methods:**

We used the full-length spike (S) protein of SARS-CoV-2 for the development of qualitative and quantitative IgG and IgA anti-S enzyme linked immunosorbent assays (ELISA). A total of 320 sera used for assay development were comprised of pandemic sera from SARS-CoV-2 infected adults (n=51) and pre-pandemic sera (n=269) including sera from endemic human coronavirus infected adults. Reverse cumulative curves and diagnostic test statistics were evaluated to define the optimal serum dilution and OD cutoff value for IgG anti-S and IgA anti-S ELISAs. The IgG and IgA anti-S, and three functional antibodies (ACE-2 receptor blocking antibody, lentipseudovirus-S neutralizing antibody, and SARS-CoV-2 neutralizing antibody) were measured using additional SARS-CoV-2 PCR positive sera (n=76) and surveillance sera (n=25). Lastly, the IgG and IgA anti-S levels were compared in different demographic groups.

**Results:**

The optimal serum dilution for the qualitative IgG anti-S ELISA was at 1:1024 yielding a 99.6% specificity, 92.2% sensitivity, 92.9% positive predictive value (PPV), and 99.6% negative predictive value (NPV) at a SARS-CoV-2 seroprevalence of 5%. The optimal serum dilution for the qualitative IgA anti-S ELISA was at 1:128 yielding a 98.9% specificity, 76.5% sensitivity, 78.3% PPV, and 98.8% NPV at the same seroprevalence. Significant correlations were demonstrated between the IgG and IgA (r=0.833 for concentrations, r=0.840 for titers) as well as between IgG and three functional antibodies (r=0.811-0.924 for concentrations, r=0.795-0.917 for titers). The IgG and IgA anti-S levels were significantly higher in males than females (p<0.05), and in adults with moderate/severe symptoms than in adults with mild/moderate symptoms (p<0.001).

**Conclusion:**

We developed a highly specific and sensitive IgG anti-S ELISA assay to SARS-CoV-2 using full length S protein. The IgG anti-S antibody level was strongly associated with IgA and functional antibody levels in adults with SARS-CoV-2 infection. Gender and disease severity, rather than age, play an important role in antibody levels.

## Introduction

In December 2019, a novel coronavirus emerged in China that has subsequently proven to be the causative agent of an acute respiratory disease now known as coronavirus disease 2019 (COVID-19) (1), and has since sparked a pandemic. The virus was officially named severe acute respiratory syndrome coronavirus 2 (SARS-CoV-2) (2). It is the seventh coronavirus species to cross the species barrier causing respiratory infections in humans (3, 4). Compared with the earlier SARS-CoV in 2003 and the Middle East Respiratory Syndrome Coronavirus (MERS-CoV) in 2012, SARS-CoV-2 has spread rapidly causing at least 201 million cases and 4.3 million deaths globally, as of August. 6^th^, 2021. Therefore, it is critical to develop mitigation strategies to control the spread of this emerging virus and use robust serological assays to determine the vulnerability of a population, define immune correlates, and evaluate vaccines.

SARS-CoV-2 is a positive-sense single-stranded RNA virus, measuring 50–200 nanometers in diameter. The virus has four structural proteins: S (spike), E (envelope), M (membrane), and N (nucleocapsid) proteins. The S protein is responsible for attaching to the host membrane-bound receptor, angiotensin-converting enzyme 2 (ACE-2), and fusing with the membrane of the host cell (5, 6). Specifically, the N-terminal S1 subunit catalyzes attachment to the host receptor, and the C-terminal S2 subunit mediates membrane fusion. The S1 subunit is further divided into the N terminal domain (NTD) and the receptor binding domain (RBD). In adults infected with SARS-CoV-2, neutralizing antibodies are generated to the S protein and its RBD, both of which are major targets for vaccine development (7). Antibodies are also generated to other structural proteins, particularly to the N protein, which is often included in commercial diagnostic antibody tests (8).

Antibody assays are valuable tools that can be utilized for diagnosing an acute infection, determining seroprevalence in a population, evaluating the immunogenicity of vaccines, studying antibody response induced by wild type infection, and establishing immune correlates of protection. Data from seroprevalence studies can be used to determine the vulnerability of a population and to identify those at risk for infection and reinfection. Sensitive and specific antibody assays that measure anti-SARS-CoV-2 antibodies are, therefore, crucial tools in the arsenal needed for gaining control of the SARS-CoV-2 pandemic. There are two major types of assays used to characterize serum antibody responses: assays that measure the presence, concentration, and isotype of antigen-specific binding antibodies; and those that measure the functional capabilities of serum antibodies based on their ability to block viral binding to cellular receptors, or neutralize viral infection. The emergency use authorization (EUA) has allowed the United States Food and Drug Administration (FDA) to provide rapid emergency use of unapproved diagnostic testing, including antibody-based assays. However, several shortcomings have been observed with the sensitivity and specificity of these tests (9, 10) perhaps, in part, because of an inadequate sample size used for assay development (11, 12). In the present study, we used the full length S protein of SARS-CoV-2 as the capture antigen and developed a qualitative and quantitative IgG anti-S ELISA assay using pandemic sera from SARS-CoV-2 infected adults (n=51) and pre-pandemic sera (n=269) including sera from endemic human coronavirus (229E, OC43, NL63, HKU1) infected adults. The IgG anti-S ELISA assay showed high specificity, sensitivity, positive predictive values (PPV) and negative predictive values (NPV) at a SARS-CoV-2 seroprevalence of 5%. Further, the developed assay was utilized for the detection of anti-S SARS-CoV-2 antibodies in SARS-CoV-2 polymerase chain reaction (PCR) positive human sera. We found that the presence and concentration of IgG anti-S was strongly associated with IgA anti-S binding antibodies, ACE-2 receptor blocking antibody activity, lentipseudovirus-S neutralization antibody concentration, and SARS-CoV-2 neutralization antibody titer. Importantly, the assay allows for a qualitative assessment (yes/no) of the presence of anti-SARS-CoV-2 antibodies as well as a quantitative measurement of antibody concentration and titer. We also discovered that the IgG and IgA anti-S levels were significantly higher in males than females, and in adults with moderate/severe symptoms than in adults with mild/moderate symptoms.

## Materials and Methods

### Serum Samples

SARS-CoV-2 assay development was performed with 320 sera collected from: (1) 234 pre-pandemic adults (before December 2019); (2) 35 pre-pandemic adults who were PCR positive for one of the four human endemic coronaviruses (229E, OC43, NL63, or HKU1); and (3) 51 SARS-CoV-2 PCR positive adults 4 to 8 weeks after their first positive PCR test. After the IgG anti-S ELISA assay was developed and optimized, an additional 25 SARS-CoV-2 PCR positive adult sera collected 4 to 8 weeks after their first PCR positive test, and 25 unknown infectious status sera from adults enrolled in a SARS-CoV-2 surveillance study were evaluated for IgG anti-S antibody responses. Demographic information was collected as well clinical disease categories, which were based on the level of care provided. Subjects with asymptomatic/mild symptoms did not require medical evaluation, subjects with mild/moderate symptoms were evaluated at the clinic or emergency department for their acute illness, and subjects with moderate/severe symptoms were hospitalized for supportive or ICU care. These additional 50 sera were also tested by additional serological assays described below following assay optimization. The median days of post-infection for these 76 SARS-CoV-2 PCR positive adults was 39 days (IQR 31-49 days). The institutional review board of Baylor College of Medicine approved the study protocols and written informed consent was obtained from all the participants. All sera were stored at -30°C until use.

### Assay Development

The IgG anti-S ELISA and other 4 serological assays were developed and optimized to quantify and/or functionally characterize serum antibodies binding to the full length spike protein of SARS-CoV-2 or to the virus. The IgG anti-S and IgA anti-S ELISAs were developed to quantify the concentrations of IgG and IgA present in the serum of subjects who had tested positive by SARS-CoV-2 PCR assay. Both relative concentration (ng/mL) and titer (log2) for anti-S antibody were determined in the two assays. The functional capabilities of anti-S antibodies were evaluated using an ACE-2 receptor blocking antibody assay, a lentipseudovirus-S neutralization assay, and a SARS-CoV-2 microneutralization assay. Only quantitative assays were developed for the three functional antibody assays. Prior to assay optimization, the ideal concentration for each of the reagents (antigen, primary and secondary antibodies, recombinant proteins, etc.) was determined as well as assay standards and positive and negative controls.

### IgG Anti-SARS-CoV-2 S (IgG Anti-S) ELISA

Immulon 2HB 96-well plates (Cat. # 3455, Thermo Scientific) were rinsed with distilled water and air dried. One hundred µL of SARS-CoV-2 full S protein (kindly provided by Gale Smith, Novavax, Gaithersburg, MD) at a concentration of 300 ng/mL in 1X Dulbecco’s Phosphate-Buffered Saline (DPBS) (Cat. # 14190-2350, Thermo Fisher) was coated onto the 96-well plate for 18 hours at 4°C. For IgG antibody detection in sera, 100 µL of human IgG immunoglobulin positive control (Cat. # 55908, MP Biomedical) at 100 ng/mL and 25 ng/mL were coated on the plates as high and low IgG positive controls, respectively. After three washes with 1X KPL (Cat. # 95059-132, VWR), the plates were blocked for 1 hour with 5% milk (Carnation Instant Nonfat Dry Milk) in 1X KPL. Two-fold serial dilutions (40 ng/mL to 0.04 ng/mL) of IgG rabbit SARS-CoV-2 S1 monoclonal antibody (mAb) (40150-R007, SinoBiological) were added to each plate to generate a relative IgG anti-S standard curve. Rabbit SARS-CoV-2 positive serum (1:150,000 in 10% FBS/5% milk/1X KPL) and human SARS-CoV-2 negative serum (1:512 in 10% FBS/5% milk/1X KPL) were used as additional assay controls. Next, 100 µL of 2-fold serial dilutions of test sera (1:32 to 1:32,768) in duplicates in 5% milk/1X KPL were added to the coated plates, followed by 1 hour incubation at 36°C. Therefore, each test plate contained its own standard curve, a high and low human IgG immunoglobulin positive control, a positive IgG rabbit serum control, a negative human serum control, and three serum test samples starting at 1:32 dilution. Plates were washed 3 times with 1X KPL after incubation. *Horseradish peroxidase (HRP)*-conjugated anti-rabbit IgG (Cat. # 170-6515, BioRad) at 1:2,000 dilution in 1X KPL was added into the wells containing the rabbit IgG SARS-CoV-2 S1 mAb and rabbit positive control serum, and HRP-conjugated anti-human IgG (Cat. # 172-1050, BioRad) at 1:2,000 dilution in 1X KPL was added into the wells containing test sera. After 1 hour incubation at 36°C, the plates were washed 6 times with 1X KPL and developed with 3,3’,5,5’-Tetramethylbenzidine (TMB) 2-Component Peroxidase Substrate (Cat. # 50-76-03, Kirkegaard and Perry Labs) for 18 min in the dark at 25°C. The reactions were stopped with 0.16 M sulfuric acid. The developed plates were read at 450 nm wavelength on a Synergy H1 microplate reader (BioTek) within 30 minutes of stopping the reaction. The SARS-CoV-2 IgG standard curve was generated from the rabbit IgG SARS-CoV-2 S1 mAb using a four-parameter logistic (4PL) regression model in Gen5 software. The relative IgG concentration (µg/mL) of test samples was determined according to the dynamic range of the standard curve by interpolating the concentration of the standards that corresponds to the absorbance value at which the test sample gave approximately half of the optical density (O.D.) of the 95% of the maximum O.D. of the standard. The IgG anti-S titer of the test samples was determined by the last dilution that gave an average O.D. value of 0.5 or greater, which was at least 3 standard deviations above the negative controls and reported in log2 value.

### IgA Anti-SARS-CoV-2 S (IgA Anti-S) ELISA

Plate rinsing and S antigen coating were the same as described above for IgG detection. Then, 100 µL of human IgA immunoglobulin positive control (Cat. # 55905, MP Biomedical) at 30 ng/mL and 7 ng/mL were coated on the plates as high and low IgA positive controls, respectively. After three washes with 1X KPL, the plates were blocked for 1 hour with 5% milk in 1X KPL. Human IgA anti-S1 mAb (Cat. # AB01680-16.0, Absolute Antibody) (2-fold serial dilutions from 70 ng/mL to 1.1 ng/mL) was added to each plate to generate an IgA anti-S standard curve. Human SARS-CoV-2 positive serum (1:250 in 10% FBS/5% milk/1X KPL) and human SARS-CoV-2 negative serum (1:512 in 10% FBS/5% milk/1X KPL) were used as assay positive and negative controls for IgA anti-S detection. Next, 100 µL of 2-fold serial dilutions of test sera (1:32 to 1:2048) in duplicates in 5% milk/1X KPL were added to the coated plates, followed by 1 hour incubation at 36°C. Therefore, each test plate contained its own standard curve, a high and low human IgA immunoglobulin positive control, a human IgA positive serum control, a negative human serum control, and five serum test samples starting at a 1:32 dilution. Plates were washed 3 times with 1X KPL after the incubation. HRP-conjugated anti-human IgA (Cat. # PA174395, Invitrogen) at 1:4,000 dilution in 1X KPL was added to all wells in the plate and incubated for 1 hour, followed by TMB color development for 10 to 18 minutes. The reaction was stopped with 0.16 M sulfuric acid and the plates were read within 30 minutes as per the IgG anti-S ELISA. The IgA concentration (µg/mL) of test samples was determined according to the dynamic range of the standard curve by interpolating the concentration of the standards that corresponds to the absorbance value at which the test sample gave approximately half of the O.D. of the 95% of the maximum O.D. of the standard. The IgA anti-S titer of the test samples was determined by the last dilution that gave an average O.D. value of 0.4 or greater, which was at least 3 standard deviations above the negative controls and reported in log2 value.

### ACE-2 Receptor Blocking Antibody Assay

A brief summary of the ACE-2 receptor blocking antibody assay is provided here to better understand the details of the assay. First the S protein is coated onto the plates, followed by the addition of serum. If the serum contains antibody to the S protein, it will bind to the recombinant S protein coated on the plate and block the subsequent binding of biotinylated recombinant ACE-2 (b-ACE2). Blocking is evidenced by a decrease in O.D. at 450 nm when compared to a serum control that does not block or a control well containing no serum (100% b-ACE2 binding).

Plate rinsing, S antigen coating, plate blocking and plate washing are the same as described above for IgG and IgA anti-S ELISAs. After aspirating the milk from plates, a SARS-CoV-2 positive human serum pool (1:32) and a SARS-CoV-2 negative human serum (1:1020) control were used as receptor blocking antibody positive and negative controls, respectively. 100 µL of 2-fold serial dilutions of test sera (1:32 to 1:2048) in duplicates in 5% milk/1X KPL were added to the S antigen coated plates. 1X DPBS instead of serum was used as a maximum b-ACE-2 binding control (no blocking control) and a b-ACE-2 negative control (HRP-conjugated streptavidin alone) (Cat. # 5270-0029, Sera Care) was also included. Then the plates were incubated for 1 hour at 25°C. The plates were washed three times with 1X DPBS. ACE-2 (kindly provided by Gale Smith, Novavax, Gaithersburg, MD) was biotinylated with a Pierce™ Antibody Biotinylation Kit (Pierce, Rockford, IL) as per manufacturer’s instructions. B-ACE-2 (2-fold serial dilutions from 1,000 ng/mL to 15.6 ng/mL) was added to generate a reference curve, and b-ACE-2 at 1 µg/mL was also added to each test serum sample to determine the serum blocking activity. The plates were incubated for 1 hour at 25°C and washed three times with 1X PBS. Then HRP-conjugated streptavidin at 1:8,000 dilution in 1X KPL was added to the plates. After 1 hour incubation at 25°C, the plates were washed 3 times with 1X KPL and color developed with TMB for 10 min in the dark at 25°C. The reactions were stopped with 0.16 M sulfuric acid and the 96-well plates were read as described above. The blocking percentage of ACE-2 receptor blocking antibody at serum dilution of 1:32 was calculated by using the following formula: [1 – (O.D._serum sample at 1:32_/O.D._maximum binding control_) x 100%]. Then the percentage was transformed to log2 values (5 log2%).

### SARS-CoV-2 Lentipseudovirus Neutralization Assay

HEK-293T cells stably expressing human angiotensin-converting enzyme 2 (hACE-2) (HEK-293T-ACE-2) (BEI, NR-52511, deposited by J. Bloom) were maintained in Dulbecco’s modified Eagle’s medium (high glucose) supplemented with 10% heat-inactivated fetal bovine serum, 2 mM L-glutamine, 1 mM sodium pyruvate, 100 U/ml penicillin, and 100 µg/ml streptomycin (DMEM complete). To generate infectious SARS-CoV-2 spike-lentiviral vector pseudotyped virions (S-PsV), 293T cells were co-transfected with pNL4-3.Luc.R-.E- (13, 14), HIV-1 gag-pol expression vector, Δ8.9, and a plasmid encoding a codon optimized SARS-CoV-2 spike protein with a C-terminal flag-tag, p278-1 (kindly provided by V. Munster, NIAID, NIH (15), using GeneJuice (Cat. # 709674, Millipore-Sigma). Two days post-transfection, the cell supernatants were harvested, passed through a 0.45 μm syringe filter, aliquoted and stored at -80°C. Infectious titers of the S-PsV stocks were determined by 2-fold endpoint dilution analysis using 293T-hACE-2 cells for infection. Two days post-infection, cells were washed once with phosphate buffered saline and lysed in Promega Glo Lysis Buffer (Cat. # E2661, Promega). Lysates were mixed with Promega Luciferase Assay Substrate (Cat. # E1501, Promega), and luminescence (relative light units/sec) was quantified in a luminometer. To perform the 293T-hACE-2 neutralization assay, 96 well tissue-culture plates were precoated with 100 µg/mL Poly-D-Lysine (Cat. # P6407, Millipore-Sigma) and incubated at room temperature for 5 minutes. Following incubation, wells were washed with tissue-culture grade water and dried. The 293T-hACE-2 cells were plated and cultured (1.5 x 10^4^ cells per well) for 24 hours. Donor sera from SARS-CoV-2 infected patients were heat-inactivated for 30 min at 56°C and serially 2.5-fold diluted with DMEM media over a range from 1:40 to 1:61,035. Diluted sera were incubated with 250-300 infectious units of pseudotyped SARS-CoV-2 indicator virus for one hour at 37°C 5% CO_2_. Dilutions were carried out in duplicates with sera free positive controls and no virus negative controls in quadruplicates. Following incubation of diluted sera and indicator virus, sera-virus mixtures were used to infect 293T-hACE-2 cells and incubated at 37°C for 48 hours. After incubation, supernatants were decanted and cells were washed with PBS. Cells were then lysed using Promega Glo Lysis Buffer. Lysates were mixed with Promega Luciferase Assay Substrate, and luminescence was quantified in a luminometer. IC_50_ was calculated by nonlinear regression using GraphPad Prism 8.

### SARS-CoV-2 Microneutralization Assay

Twelve and a half µL of 2-fold serial dilutions of test sera (1:8 to 1:16384) in duplicates in 1X Minimal Essential Medium with 2% FBS (Cat. # SH30070.03, HyClone) were added in a 96-well cell culture plate (Cat. # 353072, Corning). Two extra plates were used as assay control plates in each assay. The first control plate was “no virus” control wells containing only cells in the medium, and “virus only” control wells containing 27 TCID_100_ of SARS‐CoV‐2 isolate USA‐WA1/2020, (Cat. # NR‐52281, BEI Resources). The second control plate contained two SARS-CoV-2 negative control sera and two SARS-CoV-2 positive control sera. The four control sera were also titrated (1:8 to 1:16384). Next, 50 µL of 27 TCID_100_ of SARS‐CoV‐2 was added onto the sera. The plates were incubated for 2 hours at 36°C and 5% CO_2_. Then, 1.5 x 10^5^ Vero.E6 trypsinized cells (ATCC #CRL‐1586) were added to each well and the plates were incubated at 36°C for 3-4 days. The cells were then fixed and stained with 100 uL of a 10% Neutral Buffered Formalin/0.01% crystal violet solution for 24-48 hours. The neutralizing antibody (NtAb) titer was determined by calculating the highest dilution at which there was a 50% reduction in viral cytopathic effect. Then the dilution factor was log transformed into log2 titer. The lower limit of detection was 2.5 log2. Samples with a titer less than 2.5 were assigned a value of 2.0.

### Statistical Analysis

Diagnostic test statistics were calculated as measures of assay performance. For a given seroprevalence, PPV and NPV were estimated based on Bayes’ Theorem. Age groups, gender, and disease severity differences in log transformed geometric mean concentrations (log2 ng/mL) and geometric mean titers (log2) of IgG and IgA anti-S antibodies were analyzed by one-way ANOVA or independent Student’s t test. Pearson’s Correlation coefficients were calculated between IgG anti-S level and IgA anti-S level as well as between IgG anti-S level and functional antibody levels. Stata version 16 and SPSS version 22 were used to perform statistical analyses.

## Results

### Assay Repeatability

To demonstrate the test-retest repeatability (i.e. both reproducibility and agreement) of the IgG anti-S and IgA anti-S ELISAs, we tested 45 human sera independently three times with both of the assays. The sera tested included pre-pandemic sera (n=15), pre-pandemic sera with known human endemic coronavirus infection (229E, OC43, NL63, or HKU1) (n=15), and SARS-CoV-2 PCR positive sera (n=15). The data were computed for Intraclass Correlation Coefficient (ICC) as estimates of assay repeatability. The ICC for IgG anti-S concentration (log2 µg/mL) and titer (log2) demonstrated an excellent repeatability of 0.997 (95% CI=0.991-0.999) and 0.969 (95% CI=0.937-0.984), respectively. The IgA anti-S assay also showed an excellent repeatability, with ICC of IgA anti-S concentration (log2 ng/mL) and titer (log2) of 0.976 (95% CI=0.959-0.987) and 0.989 (95% CI=0.975-0.993), respectively.

### Assay Specificity, Sensitivity, Positive Predictive Value (PPV) and Negative Predictive Value (NPV)

**Figure 1:**
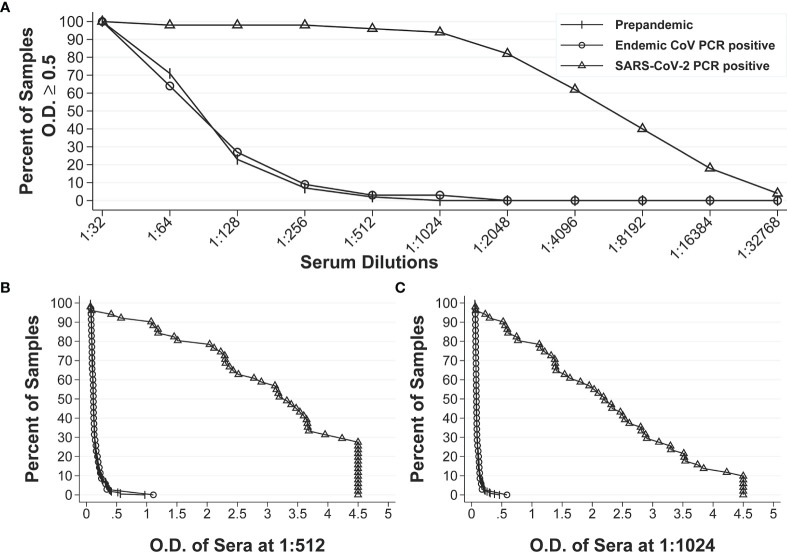
Reverse cumulative distribution curves of IgG anti-S ELISA. **(A)** Percentages of positive sera among the pre-pandemic sera with unknown infectious status to endemic coronaviruses (n = 234), pre-pandemic human endemic coronavirus PCR positive sera (n=35), and SARS-CoV-2 PCR positive sera (n=51) at different dilutions at an O.D. ≥ 0.5. **(B, C)** Percentages of positive sera at different O.D. values for all three groups of sera at 1:512 dilution and 1:1024 dilution, respectively.

After establishing the repeatability of our IgG anti-S and IgA anti-S ELISAs, we sought to further define the assays by determining the specificity, sensitivity, positive predictive value (PPV) and negative predictive value (NPV) of the assays. We used a larger panel of 320 human sera that included three groups: pre-pandemic sera with unknown infectious status to endemic coronaviruses (n=234), pre-pandemic sera with endemic human coronavirus infection (n=35), and SARS-CoV-2 PCR positive sera (n=51). A reverse cumulative distribution curve for anti-S IgG revealed that two dilutions (1:512 and 1:1,024) showed the lowest and highest percentages of positive samples for pre-pandemic sera and SARS-CoV-2 PCR positive sera, respectively at a cutoff O.D. of ≥0.5 (**Figure 1A**). That is, at dilutions of 1:512 and 1:1,024 and a cutoff O.D. of ≥ 0.5, the pre-pandemic sera had the lowest percent positive samples and the SARS-CoV-2 positive samples had the highest percentage of positive samples, thus indicating high specificity and sensitivity. We then performed diagnostic test statistics to determine the optimal dilution and O.D. cutoff that would result in the highest specificity and sensitivity. For serum diluted 1:512, an O.D. of 0.5 resulted in specificity of 98.1% and sensitivity of 94.1% (**Figure 1B**). For serum diluted 1:1,024, an O.D. of 0.5 resulted in specificity of 99.6% and sensitivity of 92.2% (**Figure 1C**). In addition, the 1:1,024 serum dilution resulted in higher PPV (92.9%) than that of the 1:512 dilution (72.7%) at a seroprevalence of 5% (**Table 1**), while the NPVs remained similar (99.6% *vs* 99.7%) for both dilutions at the same seroprevalence. Therefore, the 1:1,024 dilution and O.D.≥0.5 were determined to be the final dilution factor and cutoff O.D. values for determining the IgG anti-S positivity of a given sample.

**Table 1:** Positive predictive value (PPV) and negative predictive value (NPV) of SARS-CoV-2 IgG at different SARS-CoV-2 seroprevalence rates.

SARS-CoV-2	1:512 Dilution		1:1,024 Dilution	
Seroprevalence Rates	PPV % (95% CI)	NPV % (95% CI)	PPV % (95% CI)	NPV % (95% CI)
1%	33.8% (17.6, 55)	99.9% (99.8, 100)	71.5% (26.1, 94.7)	99.9% (99.8, 100)
**5%**	72.7% (52.7, 86.4)	99.7% (99.1, 99.9)	**92.9% (64.8, 98.9)**	**99.6% (98.9, 99.8)**
7.5%	80.4% (63.2, 90.7)	99.5% (98.6, 99.8)	95.3% (73.9, 99.3)	99.4% (98.4, 99.8)
10%	84.9% (70.2, 93.1)	99.3% (98, 99.8)	96.5% (79.5,99.5)	99.1% (97.8, 99.7)
15%	89.9% (78.9, 95.5)	99% (96.9, 99.6)	97.8% (86.1, 99.7)	98.6% (99.6, 99.5)
20%	92.7% (84.1, 96.8)	98.5% (95.7, 99.5)	98.4% (89.7, 99.8)	98.1% (95.2, 99.2)
50%	98.1% (95.5, 99.2)	94.3% (84.8, 98)	99.6% (97.2, 99.9)	92.7% (83.2, 97)

A Bayesian approach was used to estimate the PPV and NPV for a given prevalence. The bold values they are the final numbers reported in the Abstract. They are the determined/optimal PPV and NPV that helped to determine the optimal dilution factors for IgG assay (1:1024) and IgA assay (1:128) at the seroprevalence of 5% calculated with the 320 assay development samples.

**Figure 2:**
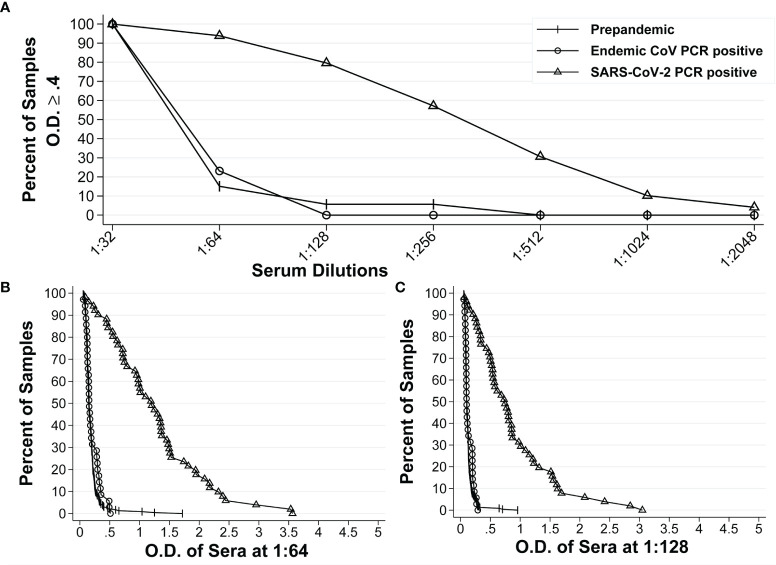
Reverse cumulative distribution curves of IgA anti-S ELISA. **(A)** Percentages of positive sera among the pre-pandemic sera with unknown infectious status to endemic coronaviruses (n = 234), pre-pandemic human endemic coronavirus PCR positive sera (n=35), and SARS-CoV-2 PCR positive sera (n=51) at different dilutions at an O.D. ≥ 0.4. **(B, C)** Percentages of positive sera at different O.D. values for all three groups of sera at 1:64 dilution and 1:128 dilution, respectively.

We also calculated these parameters for the IgA anti-S ELISA. The reverse cumulative distribution curve for IgA anti-S showed that two dilutions (1:64 and 1:128) had the lowest and highest percentages of positive samples for pre-pandemic sera and SARS-CoV-2 PCR positive sera, respectively at a cutoff O.D. of ≥0.4 (**Figure 2A**). For serum diluted 1:64, an O.D. of 0.4 resulted in specificity of 97.8% and sensitivity of 84.3% (**Figure 2B**). For serum diluted at 1:128, an O.D. of 0.4 resulted in specificity of 98.9% and sensitivity of 76.5% (**Figure 2C**). In addition, the 1:128 dilution resulted in higher PPV (78.3%) than that of 1:64 dilution (66.5%) at serum prevalence of 5% (**Table 2**), while the NPVs remained similar (98.8% *vs*. 99.2%) for both dilutions at the same serum prevalence. Therefore, the 1:128 dilution and O.D.≥0.4 were determined to be the final dilution factor and cutoff O.D. values for determining the IgA anti-S positivity of a given sample.

**Table 2:** Positive predictive value (PPV) and negative predictive value (NPV) of SARS-CoV-2 IgA at different SARS-CoV-2 seroprevalence rates.

SARS-CoV-2	1:64 Dilution		1:128 Dilution	
Seroprevalence Rates	PPV % (95% CI)	NPV % (95% CI)	PPV % (95% CI)	NPV % (95% CI)
1%	18.2% (11.0, 28.6)	99.9% (99.8, 100)	40.9% (18.2, 68.3)	99.8% (99.6, 99.9)
**5%**	53.7% (39.3, 67.6)	99.5% (98.8, 99.8)	**78.3% (53.7, 91.8)**	**98.8% (98.0, 99.2)**
7.5%	64.1% (49.9, 76.3)	99.2% (98.1, 99.6)	84.8% (64.1, 94.5)	98.1% (96.9, 98.8)
10%	71.0% (57.7, 81.5)	98.9% (97.5, 99.5)	88.4% (71.0, 96.0)	97.4% (95.8, 98.4)
15%	79.6% (68.4, 87.5)	98.2% (96.0, 99.2)	92.4% (79.5, 97.4)	96.0% (93.6, 97.5)
20%	84.6% (75.4, 90.8)	97.5% (94.4, 98.9)	94.5% (84.6, 98.2)	94.4% (91.1, 96.5)
50%	95.7% (92.5, 97.5)	90.7% (81, 95.7)	98.6% (95.7, 99.5)	80.8% (71.9, 87.3)

A Bayesian approach was used to estimate the PPV and NPV for a given prevalence. The bold values they are the final numbers reported in the Abstract. They are the determined/optimal PPV and NPV that helped to determine the optimal dilution factors for IgG assay (1:1024) and IgA assay (1:128) at the seroprevalence of 5% calculated with the 320 assay development samples.

In addition to the above PPV and NPV based on the seroprevalence of 5% in the early months of the pandemic (June 2020), we also calculated the PPV and NPV of IgG anti-S and IgA anti-S ELISAs at a seroprevalence of 50%, which is closer to the seroprevalence that is occurring now (August 2021) in much of the United States. The PPV and NPV of the IgG anti-S ELISA assay at 1:512 dilution was 98.1% (95.5, 99.2) and 94.3% (84.8, 98), respectively, and at 1:1,024 dilution was 99.6% (97.2, 99.9), and 92.7% (83.2, 97), respectively (**Table 1**). The PPV and NPV of the IgA anti-S ELISA assay at 1:64 dilution was 95.7% (92.5, 97.5) and 90.7% (81, 95.7), respectively, and at 1:128 dilution was 98.6% (95.7, 99.5) and 80.8% (71.9, 87.3), respectively (**Table 2**).

### SARS-CoV-2 PCR Positive and Surveillance Subjects

**Table 3** shows the demographic information of the 101 subjects who donated their pandemic sera prior to June 3^rd^, 2020. The cohort include 76 SARS-CoV-2 PCR positive subjects and 25 surveillance subjects and were used in all assays except for SARS-CoV-2 lentipseudovirus neutralization assay (73 SARS-CoV-2 PCR positive subjects, 6 surveillance subjects) due to the limited volumes of sera. The demographic information for these 101 subjects included the numbers and percentages by age groups, gender, race, ethnicity, co-morbid conditions, occupation, hospitalization, and disease severity. The 76 SARS-CoV-2 PCR positive subjects included 1). 40 male (median age 49 years, IQR 39-62 years) and 36 female (median age 46 years, IQR 35-58 years); 2). 14 subjects were hospitalized and 62 subjects were not; 3). 2 subjects with asymptomatic/mild symptoms (did not require medical evaluation), 60 subjects with mild/moderate symptoms (evaluated at the clinic or emergency department for their acute illness), and 14 subjects with moderate/severe symptoms (were hospitalized for supportive or ICU care). The 25 unknown SARS-CoV-2 infection status from adults enrolled in our SARS-CoV-2 surveillance study included 1) 9 male (median age 45 years, IQR 30-54 years) and 16 female (median age 39 years, IQR 29-47 years); 2). 22 subjects with asymptomatic/mild symptoms and 3 subjects with mild/moderate symptoms; and 3) none of the subjects were hospitalized.

**Table 3:** Demographic data of the subjects during SARS-CoV-2 pandemic.

Variables	# of SARS-CoV-2 PCR positive subjects (%)	# of surveillance subjects (%)	# of both subjects (%)
**Age (years)**			
18-34	16 (21.1)	9 (36)	25 (24.8)
35-49	27 (35.5)	10 (40)	37 (36.6)
50-64	22 (28.9)	4 (16)	26 (25.7)
≥ 65	11 (14.5)	2 (8)	13 (12.9)
**Gender**			
Male	36 (47.4)	9 (36)	45 (44.6)
Female	40 (52.6)	16 (64)	56 (55.4)
**Race**			
White	50 (65.8)	18 (72)	68 (67.3)
Black	7 (9.2)	1 (4)	8 (7.9)
Asian	14 (18.4)	2 (8)	16 (15.8)
American Indian or Alaska Native	0 (0.0)	2 (8)	2 (2.0)
Multiracial	3 (3.9)	1 (4)	4 (4.0)
Declined	2 (2.6)	1 (4)	3 (3.0)
**Ethnicity**			
Hispanic	15 (19.7)	8 (32)	23 (22.8)
Non-Hispanic	61 (80.3)	17 (68)	78 (77.2)
**Co-morbid conditions**			
0	45 (59.2)	15 (60)	60 (59.4)
1	17 (22.4)	9 (36)	26 (25.7)
2	12 (15.8)	1 (4)	13 (12.9)
≥3	2 (2.6)	0 (0)	2 (2.0)
**Occupation**			
Healthcare	24 (31.6)	10 (40)	34 (33.7)
Non-healthcare	52 (68.4)	15 (60)	67 (66.3)
**Hospitalized**			
Yes	14 (18.4)	0 (0)	14 (13.9)
No	62 (81.6)	25 (100)	87 (86.1)
**Disease Severity**			
Asymptomatic/Mild	2 (2.6)	22 (88)	24 (23.8)
Mild/Moderate	60 (79)	3 (12)	63 (62.4)
Moderate/Severe	14 (18.4)	0 (0)	14 (13.9)

for the co-morbid conditions, 0=none, 1=one co-morbid condition, 2=two co-morbid conditions, ≥3=three or more co-morbid conditions.

### Comparison of Positivity and Negativity of IgG Anti-S to IgA Anti-S, Blocking Antibody, Lentipseudovirus-S NtAb, and SARS-CoV-2 NtAb

For the IgG anti-S negative sera, the percent agreement of the negative results for IgA anti-S, SARS-CoV-2 blocking Ab, lentipseudovirus-S NtAb, and SARS-CoV-2 NtAb were 93.3%, 86.7%, 93.3%, and 96.7%, respectively. For the IgG anti-S positive sera, the percent agreement of the positive results for these 4 assays were 77.5%, 95.8%, 100%, and 98.6%, respectively (**Table 4**). Therefore, we obtained a strong and consistent agreement between IgG anti-S and the other 4 antibody data. Conversely, IgA anti-S were positive for 2 sera among the 30 IgG anti-S negative sera. These data suggest that the IgA anti-S ELISA assay can be used as a secondary confirmation of seropositivity, and the combination of both IgG and IgA anti-S ELISAs will likely enhance the ability to detect a true SARS-CoV-2 infection, particularly in adults who have low levels of IgG anti-S binding antibodies.

**Table 4:** Percent agreement of positivity and negativity of IgA binding and functional antibodies to IgG anti-S binding antibody.

		IgG Anti-S	
		Negative (titer <10 log2)	Positive (titer ≥10 log2)
**IgA Anti-S**	Negative (titer < 7 log2)	28/30 (93.3%)	16/71 (22.5%)
	Positive (titer ≥ 7 log2)	2/30 (6.7%)	55/71 (77.5%)
**SARS-CoV-2 Blocking Ab**	Negative (5 log2% = 0)	26/30 (86.7%)	3/71 (4.2%)
	Positive (5 log2% > 0)	4/30 (13.3%)	68/71 (95.8)
**Lentipseudovirus-S NtAb**	Negative (IC_50_ = 0)	14/15 (93.3%)	0/64 (0%)
	Positive (IC_50_ > 0)	1/15 (6.7%)	64/64 (100%)
**SARS-CoV-2 NtAb**	Negative (titer ≤ 2 log2)	29/30 (96.7%)	1/71 (1.4%)
	Positive (titer > 2 log2)	1/30 (3.3%)	70/71 (98.6%)

### Correlation Between IgG and IgA Anti-S Binding Antibody Levels

**Figure 3:**
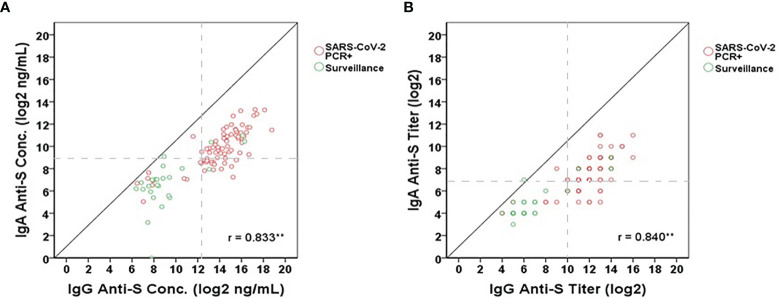
Correlation between IgG anti-S and IgA anti-S **(A)** concentrations (log2 ng/mL) and **(B)** titers (log2) in SARS-CoV-2 PCR positive (red open circles) and pandemic surveillance sera (green open circles). Gray dash lines represent the cut-offs of antibody titers (log2) or concentrations (log2 ng/mL). Pearson’s correlation coefficient was calculated to measure the strength of the linear association. *Correlation was significant at the 0.05 level (2-tailed). **Correlation was significant at the 0.01 level (2-tailed). A total of 101 serum samples including 76 from SARS-CoV-2 PCR positive adults and 25 from adults who participated in pandemic surveillance study were tested by each assay.

We used the IgA anti-S ELISA as a confirmatory test for the IgG anti-S ELISA. As part of this analysis, the correlation between the two assays was determined. Pearson’s correlation coefficient was calculated to measure the strength of the linear association between the IgG anti-S and IgA anti-S levels for the combined populations of SARS-CoV-2 PCR positive (n=76) and pandemic surveillance adults (n=25). We observed a significant positive correlation between IgG anti-S and IgA anti-S serum concentration (r=0.833, 95% CI=0.780-0.885, p<0.01) (**Figure 3A**) and antibody titer (r=0.840, 95% CI=0.775-0.894, p<0.01) (**Figure 3B**).

### Correlation Between IgG Anti-S Binding Antibody Levels and Functional Antibody Levels

**Figure 4:**
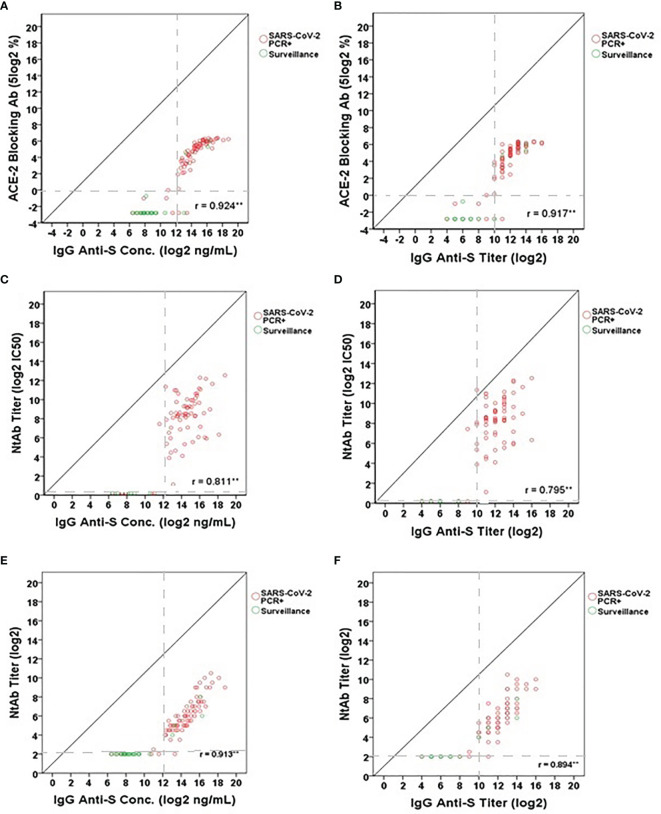
Correlation between IgG anti-S levels and **(A, B)** ACE-2 receptor blocking antibody levels (5 log2%), **(C, D)** lentipseudovirus-S neutralizing antibody titers (log2 IC_50_) and **(E, F)** SARS-CoV-2 neutralizing antibody titers (log2). A total of 101 serum samples including 76 SARS-CoV-2 PCR positive sera and 25 SARS-CoV-2 surveillance sera were tested in the IgG anti-S ELISA, ACE-2 receptor blocking antibody assay, and SARS-CoV-2 microneutralization assay. A total of 79 serum samples (73 SARS-CoV-2 PCR positive sera and 6 SARS-CoV-2 surveillance sera) were tested in the lentipseudovirus-S neutralizing antibody assay. SARS-CoV-2 PCR positive sera represented by red open circles and SARS-CoV-2 surveillance sera by green open circles. Gray dash lines represent the cut-offs of antibody titers (log2) or concentrations (log2 ng/mL). Pearson’s correlation coefficient was calculated to measure the strength of the linear association. *Correlation was significant at the 0.05 level (2-tailed). **Correlation was significant at the 0.01 level (2-tailed).

While the presence of IgG and IgA anti-S binding antibodies in SARS-CoV-2 PCR positive adults and in some of the adults participating in the SARS-CoV-2 surveillance study was indicative of a SARS-CoV-2 infection, the question remained regarding the association of ELISA binding antibodies to functional antibodies. To answer this, three different functional antibody assays were used to assess their association: ACE-2 receptor blocking antibody assay, SARS-CoV-2 lentipseudovirus-S neutralization assay, and SARS-CoV-2 microneutralization assay. Significant positive correlations were observed for all three functional antibody assays (**Figures 4A–F**). The correlations ranged from 0.811 to 0.924 (95% CI: 0.703-0.948) between IgG anti-S concentration and the functional antibody levels (**Figures 4A, C, E**) and from 0.795 to 0.917 (95% CI: 0.681-0.943) between IgG anti-S titers and the functional antibody levels (**Figures 4B, D, F**). We found that all correlations were significant (p<0.01). In addition, functional antibody activity measured by any of the three assays was not appreciable until the IgG anti-S antibody titer reached 10 log2 or greater; the threshold point used to identify serological evidence of SARS-CoV-2 infection.

### Comparison of Anti-SARS-CoV-2 Antibody Levels in Different Demographic Groups

**Figure 5:**
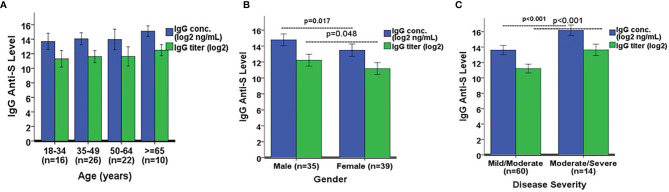
Comparison of IgG anti-S geometric mean levels by **(A)** age, **(B)** gender, and **(C)** disease severity. A one-way ANOVA was used for the statistical analysis of mean differences between age comparison, and an independent Student’s t test was employed for mean differences in gender and disease severity. A total of 74 SARS-CoV-2 PCR positive sera was analyzed in the comparison. Error bars represent standard deviations.

**Figure 6:**
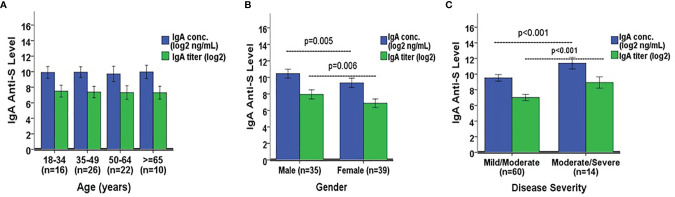
Comparison of IgA anti-S geometric mean levels by **(A)** age, **(B)** gender, and **(C)** disease severity. A one-way ANOVA was used for the statistical analysis of mean differences between age comparison, and an independent Student’s t test was employed for mean differences in gender and disease severity. A total of 74 SARS-CoV-2 PCR positive sera were analyzed in the comparison. Error bars represent standard deviations.

We next wanted to determine if there were significant differences in IgG and IgA anti-S levels among several demographic variables in the SARS-CoV-2 PCR positive adults. The only two COVID-19 asymptomatic subjects were omitted due to the small sample size. The remaining 74 subjects were analyzed here. IgG anti-S concentration and titer were similar among four separate age groups: 18-34 (n=16), 35-49 (n=26), 50-64 (n=22), and ≥65 years (n=10) (**Figure 5A**). However, the IgG anti-S levels were significantly higher in males (n=35) *versus* females (n=39) (p=0.017 for concentration, p=0.048 for titer, **Figure 5B**). The IgG anti-S levels were also significantly higher in adults with moderate/severe symptoms (n=14) than in adults with mild/moderate symptoms (n=60) (p<0.001, **Figure 5C**). Similarly, we also observed significantly higher IgA anti-S antibody levels in males and in adults with moderate/severe symptoms, but not among different age groups (**Figures 6A–C**). We also determined if there were significant differences in the three functional antibody levels by age, gender and disease severity in the SARS-CoV-2 PCR positive adults. Each of the three functional antibodies was similar among the four age groups. However, they were all higher, although not always statistically significant, in males *versus* females (p=0.052, p=0.452, p=0.042 for ACE-2 receptor blocking antibody, SARS-CoV-2 NtAb, and SARS-CoV-2 lentipseudovirus-S NtAb, respectively). Lastly, each of the three functional antibody levels were higher, although not always statistically significant, in adults with moderate/severe symptoms than in adults with mild/moderate symptoms (p<0.001, p<0.001, p=0.061 for ACE-2 receptor blocking antibody, SARS-CoV-2 NtAb, and SARS-CoV-2 lentipseudovirus-S NtAb, respectively). The three functional antibody data were generally consistent with the IgG and IgA anti-S antibody data. Similar to observation in IgG and IgA anti-S antibodies, the three functional antibody levels were observed to be higher in males and in adults with moderate/severe symptoms.

## Discussion

Human coronavirus SARS-CoV-2 has resulted in a pandemic characterized by significant physical and mental health consequences, socioeconomic stress, and decreased quality of life (16, 17). Implementation of public health efforts in controlling a pandemic requires highly sensitive and specific diagnostic and serological assays. Diagnostic assays focus on the detection of an ongoing infection. Antibody assays allow us to diagnose and track SARS-CoV-2 positive individuals, determine seroprevalence in the community, study antibody responses induced by wild type infection, evaluate the immunogenicity of vaccines, and establish immune correlates of protection. Serological assays with high specificity in order to avoid false-positive results and high sensitivity to avoid false-negative results are critical in the assessment of the ongoing SARS-CoV-2 pandemic. Thus far, the United States Food and Drug Administration (FDA) has issued EUA for about 10 ELISA assays for COVID-19 testing in the clinical health care and commercial settings in the United States (18). These assays demonstrate high specificity (96.4%-100%) and NPVs (99.5-100%), but a relatively wide range of sensitivity (90.0%-100%) and low PPVs (59.3%-100%) at 5% seroprevalence, which can be problematic (18–23).

Our IgG anti-S ELISA was developed using a cohort of 320 sera, including pre-pandemic sera, SARS-CoV-2 PCR positive sera, and sera from adults with confirmed human endemic coronavirus infections (229E, OC43, NL63, HKU1). Human endemic coronaviruses are second only to rhinoviruses as a cause of the common cold (24, 25). Antibody cross-reactivity resulting from infection with human endemic coronaviruses is of particular importance in the development of SARS-CoV-2 binding and functional antibody assays. The IgG anti-S ELISA binding assay demonstrated that sera at a dilution of 1:1,024 resulted in 99.6% specificity, 92.2% sensitivity, 92.9% PPV, and 99.6% NPV for the detection of a recent or past SARS-CoV-2 infection at a low seroprevalence of 5%. Overall, our IgG anti-S ELISA assay was comparable to those considered high performing SARS-CoV-2 antibody assays with EUA. The IgA anti-SARS-CoV-2 S ELISA had lower sensitivity and specificity than the IgG anti-S assay, however, IgA anti-S levels showed significant correlation to IgG anti-S levels in the study. Therefore, the IgA anti-S assay can be used as a confirmatory test for IgG anti-S, especially with sera having IgG antibody levels in the lower limit of detection.

An assay’s ability to detect a true positive (PPV) is highly dependent on the prevalence of the disease. The PPV and NPV of test results depends on the performance characteristics of the test (sensitivity and specificity) and on the prevalence rate of the disease in the population tested. High quality serological assays normally have PPV and NPV above 90% at a 5% seroprevalence. The NPV tends to remain stable over a wide range of seroprevalence rates in assays with high specificity. On the other hand, the PPV is greatly affected by changes in seroprevalence. We estimated the PPV and NPV of the IgG anti-S ELISA with 99.6% specificity and 92.2% sensitivity across a spectrum of SARS-CoV-2 seroprevalence rates. When the seroprevalence of SARS-CoV-2 infection in a population decreased from 5% to 1%, the PPV of the test at serum dilution of 1:1024 decreased from 92.9% to 71.5% and the NPV increased from 99.6% to 99.9%. However, as the seroprevalence increased from 5% to 20% to 50%, the PPVs also increased from 92.9% to 98.4% to 99.6%, but the NPVs decreased slightly from 99.6% to 98.1% to 92.7%. The IgA anti-S ELISA did not achieve comparable assay performance in identifying a true positive sample as compared to the IgG anti-S ELISA. The PPV of the IgA anti-S ELISA was 78.3% at a 5% seroprevalence and serum dilution of 1:128, even though the NPV remained above 90% throughout a SARS-CoV-2 seroprevalence range of 1 to 20%. At a seroprevalence of 50% and serum dilution of 1:128, the PPV increased to 98.6% but the NPV decreased to 80.8%; however, at the same seroprevalence of 50% and serum dilution of 1:64 instead of 1:128, the PPV increased to 95.7% and the NPV slightly decreased to 90.7% highlighting how the assay predictive performance changes with different seroprevalence rates and sample dilution factor. Serosurveillance studies should use assays with high specificity and PPV, especially when the prevalence of SARS-CoV-2 in the community is expected to be low. The combination of both IgG and IgA anti-S ELISAs will likely enhance the ability to detect a true SARS-CoV-2 infection, particularly in adults who have low levels of IgG anti-S binding antibodies.

We obtained a strong agreement between IgG anti-S and IgA anti-S positive sera. Fifty five out of the 71 (77.5%) IgG positive sera (from patients with mild/moderate or moderate/severe symptoms except for two patients with asymptomatic/mild disease) were positive for IgA. Besides, there was a significant positive correlation between IgG and IgA levels (r=0.833 for concentrations, and r=0.840 for titers). The strong agreement and the significant correlation suggest that the IgA anti-S ELISA assay can be used as a possible secondary confirmation of seropositivity. In addition, IgA anti-S were positive for 2 sera (from patients with asymptomatic/mild disease) among the 30 IgG anti-S negative sera, suggesting that the combination of both IgG and IgA anti-S ELISAs will likely enhance the ability to detect a true SARS-CoV-2 infection, particularly in adults who have low levels of IgG anti-S binding antibodies, as previously observed (26).

The IgG anti-S binding antibodies generated in adults with primary SARS-CoV-2 infection were highly associated with functional antibody activity as measured by the ACE-2 receptor blocking antibody assay, SARS-CoV-2 lentipseudovirus-S neutralization assay, and SARS-CoV-2 microneutralization assay. Both the ACE-2 receptor blocking antibody assay and the lentipseudovirus-S neutralization assay have the advantage of providing functional SARS-CoV-2 antibody data without having to use a BSL-3 facility. Functional antibody activity was generally not detected until IgG anti-S antibodies reached the 1:1024 antibody titer threshold. Antibodies detected by the IgG anti-S ELISA at levels below 1:1024 were likely the result of cross-reactive antibodies from past human endemic coronavirus infections. Our findings are consistent with those from other studies (27–31), and demonstrates that humoral IgG anti-S antibody is a sensitive marker of infection status and SARS-CoV-2 neutralizing activity.

A number of studies have demonstrated significant associations between SARS-CoV-2 antibody levels with host and disease variables such as age, gender, and severity of disease. In terms of seroprevalence corresponding to a specific demographic variable, it seems that locality and sample size can be highly relevant to the outcome (32). Differences in seropositivity may also be attributed to viral load (33). In our study, males had significantly higher IgG and IgA anti-S binding antibodies, consistent with results from other studies (34, 35), as did adults with more severe disease (11, 36–38). There are studies however, that demonstrate female patients more likely than male patients to generate a relatively high concentration of serum IgG anti-SARS-CoV-2 antibody in severe infection (39). The differences based on gender may be multifactorial and accounted for in part by severity of the disease, sample size, detection methods, and other host factors. Consistent with previous reports, we found that age among adults did not significantly impact the magnitude of the serum IgG anti-S antibody response (28). One notable exception is the comparison between hospitalized adults to hospitalized children where the adults had higher anti-SARS-CoV-2 antibody levels than the children (40).

In conclusion, we report an IgG anti-S ELISA assay that was developed as both a quantitative and qualitative IgG anti-S antibody assay amenable to high throughput anti-SARS-CoV-2 antibody testing. The IgG anti-S ELISA assay had excellent performance characteristics, and was strongly associated with functional antibody activity in adults with primary SARS-CoV-2 infection. Gender and disease severity, rather than, age, played a role in antibody levels. This assay will be instrumental for patient contact tracing, seroprevalence studies, and vaccine evaluation studies. The IgA anti-S ELISA assay can be used as a possible secondary confirmation of seropositivity, and provide insight into the composition of anti-SARS-CoV-2 sera.

## Data Availability Statement

The original contributions presented in the study are included in the article. Further inquiries can be directed to the corresponding author.

## Ethics Statement

The studies involving human participants were reviewed and approved by the institutional review board of Baylor College of Medicine. The patients/participants provided their written informed consent to participate in this study.

## Author Contributions

XY and PP designed the research. XY, OI, LSA, WC, LOA, TM, ZM, HL, KT, BL, MC, and PP performed the research. XY, LF-S, and PP analyzed the data. EN, NB, SF, and PS provided the samples. YR processed the samples. All authors edited the text. XY wrote the first draft of the manuscript. All authors contributed to the article and approved the submitted version.

## Conflict of Interest

The authors declare that the research was conducted in the absence of any commercial or financial relationships that could be construed as a potential conflict of interest.

## Publisher’s Note

All claims expressed in this article are solely those of the authors and do not necessarily represent those of their affiliated organizations, or those of the publisher, the editors and the reviewers. Any product that may be evaluated in this article, or claim that may be made by its manufacturer, is not guaranteed or endorsed by the publisher.
